# Fatty acid synthase cooperates with protrudin to facilitate membrane outgrowth of cellular protrusions

**DOI:** 10.1038/srep46569

**Published:** 2017-04-21

**Authors:** Chuanling Zhang, Jiaqi Lu, Huizhong Su, Jing Yang, Demin Zhou

**Affiliations:** 1 State Key Laboratory of Natural and Biomimetic Drugs, School of Pharmaceutical Sciences, Peking University, Beijing 100191, China

## Abstract

Cellular protrusion formation capacity is a key feature of developing neurons and many eukaryotic cells. However, the mechanisms underlying membrane growth in protrusion formation are largely unclear. In this study, photo-reactive unnatural amino acid 3-(3-methyl-3H-diazirin-3-yl)-propamino-carbonyl-Nε-l-lysine was incorporated by a genetic code expansion strategy into protrudin, a protein localized in acidic endosomes and in the endoplasmic reticulum, that induces cellular protrusion and neurite formation. The modified protrudin was used for covalent trapping of protrudin-interacting proteins in living cells. Fatty acid synthase (FASN), which synthesizes free fatty acids, was identified to transiently interact with protrudin. Further characterization revealed a unique cooperation mechanism in which protrudin cooperates with FASN to facilitate cellular protrusion formation. This work reveals a novel mechanism involved in protrusion formation that is dependent on transient interaction between FASN and protrudin, and establishes a creative strategy to investigate transient protein-protein interactions in mammalian cells.

Cellular protrusions are deformations that form at the surface of living cells under certain biological conditions such as neurite outgrowth^1^, cell migration, and defense^2^,^3^, providing the cell with the capacity to obtain nutrients, to movie, to sense, or respond to external physical or chemical stimuli. Protrusion formation is a result of cooperation between the plasma membrane and the underlying actin cytoskeleton. Cytoskeleton remodeling involves local severing of the long and stable microtubules (MT) of the parent axon into short highly mobile pieces that are then able to move into the newly formed branch sites to promote neurite outgrowth^4^,^5^. However, the precise architecture, function, and molecular mechanisms underlying regulation of membrane protrusion remain poorly understood.

Protrudin (synonym ZFYVE27), a protein localized in acidic endosomes and in the endoplasmic reticulum, encoded by the gene *SPG33*, previously reported to be mutated (p.G191V) in a single family with an autosomal dominant form of hereditary spastic paraplegia, induces neurite formation by directional membrane trafficking^6^,^7^,^8^. Interestingly, over-expression of protrudin in non-neuronal cells also induces formation of neurite-like membrane protrusions^1^,^9^,^10^. Moreover, recent research shows that protrudin promotes the repair of kidney injury through activation of proliferation, migration, and morphogenesis to generate multiple long tubular segments^11^. These results suggest that protrudin plays an important role in plasma membrane remodeling in a manner not previously described. To elucidate the precise function of protrudin, traditional yeast two-hybrid or immunoprecipitation (IP) experiments showed that protrudin regulates intracellular transport through permanent interactions with proteins such as atlastin, KIF5, spastin, FKBP38, and VAMP^8^,^10^,^12^,^13^,^14^. However, the precise mechanism by which protrudin modulates membrane trafficking is still unclear owing to the complexity of the dynamic process of vesicular transport.

Obviously, it is not enough to study permanent protein-protein interactions (PPIs) only. Transient PPIs frequently play essential roles in regulating intracellular dynamic processes such as vesicle transport, signal transduction, transcriptional activation, and cell cycle transition^15^. In most cases, transient PPIs are weak interactions, with Kd > μM and a short half-life in the range of minutes or seconds^15^,^16^. Elucidation of these interactions is crucial for unraveling signaling networks and for the development of therapeutic PPI inhibitors^17^. Owing to their short half-life, detection and analysis of transient protein complexes remain challenging^18^. Moreover, membrane trafficking from endocytosis to exocytosis in mammalian cells involves pH-dependent PPIs^19^. The pH declines from early sorting to late endosomes and then to lysosomes during endocytosis, and this decline is required for the redistribution and degradation of coated proteins in endosomes^20^,^21^. The acidic pH (pH < 5.5) always results in receptor–ligand disassociation, which makes the capture of this interaction much more difficult when using traditional techniques such as yeast two-hybrid screening.

To unravel the mechanisms of protrudin modulated membrane growth of cellular protrusion through a novel perspective, we employed a photo-crosslinking amino acid DiZPK (3-(3-methyl-3H-diazirin-3-yl)-propamino-carbonyl-Nε-l-lysine) and a genetic code expansion strategy for the covalent trapping of transient PPIs in a native physiological context^22^,^23^ (Fig. 1E, Supplementary Fig. 1). Our results identified fatty acid synthase (FASN) and protrudin as a new PPI that facilitates cellular protrusion in HeLa cells. Furthermore, we present a facile platform for investigating transient PPIs, especially those occurring under extreme physiological conditions.

## Results

### Use of genetic code expansion for precision engineering of the protrudin protein

**Figure 1:**
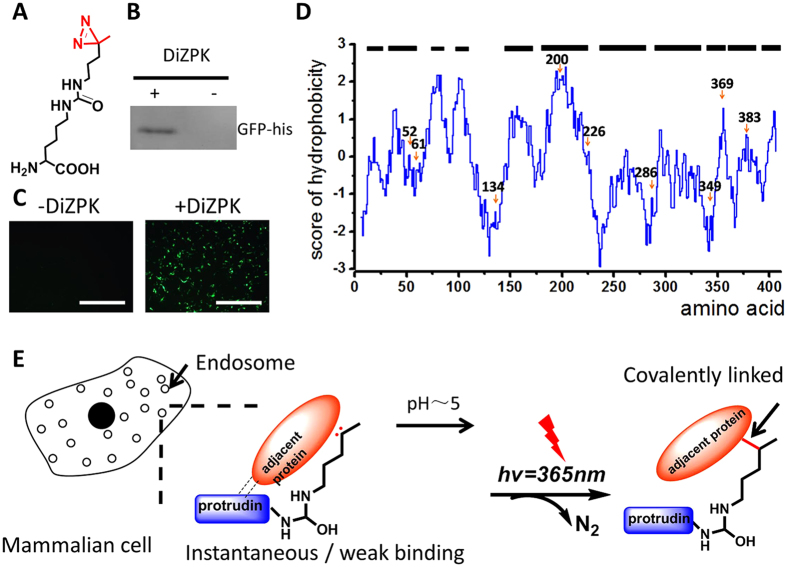
Development of DiZPK as a protein photo-crosslinking agent. (**A**) Structure of DiZPK with the photo-activation group shown in red. (**B**) Western blotting analysis showing the full-length green fluorescent protein (GFP) produced only when DiZPK was present. (**C**) The full-length GFP was detected by a fluorescence microscope in the presence of DiZPK. Scale bars, 150 μm. (**D**) Hydrophobicity analysis of protrudin. The hydrophobic sites (red arrows) of the protein were selected for DiZPK incorporation. (**E**) Scheme for capturing transient binding of proteins (in orange) with protrudin in 293T cells at approximately pH 5 using DiZPK as the photo-crosslinking probe. The pH value of the endosome is approximately 5.

To investigate the compatibility of the amber suppressor (TAG) with the orthogonal MbPylRS/MbtRNA_CUA_ pair for protein expression in mammalian cells, we first built a green fluorescent protein (GFP)-based system capable of testing the incorporation efficiency of DiZPK. A GFP-expressing vector carrying a stop codon (TAG) at position 39 and a vector carrying the gene for orthogonal MbPylRS/MbtRNA_CUA_ were co-transfected into HEK293T cells. The full-length GFP was obtained with 1 mM DiZPK in 293T cells, as observed in the western blotting and fluorescence analyses (Fig. 1B and C, respectively). No GFP expression was detected by western blotting in the absence of DiZPK, indicating that DiZPK incorporation was very specific in mammalian cells.

After successfully incorporated DiZPK into the GFP protein, we tried to incorporate it into the protrudin protein. First, the protrudin gene was cloned into the pcDNA3.1-myc vector with a myc tag at the C-terminus. Next, the protrudin-coding plasmid pcDNA3.1-protrudin-myc was mutated separately in the codons encoding residues L13, I49, E52, D56, V61, D134, G191, P200, N209, Y226, T265, T271, S274, E286, F287, D289, E292, D294, R330, T349, V352, S358, R369, G381, T383, E386, and F393, changing each codon to the amber codon (TAG) (Supplementary Table 1). The choice of codons was based on the location, involvement in known protein binding^10^,^24^, phosphorylation^24^, and hydrophobicity of the corresponding residues analyzed using online estimators with the algorithms of Kyte and Doolittle (http://web.expasy.org/cgi-bin/protscale/protscale.pl). The mutant plasmids were tested in parallel to determine whether DiZPK, a diazirine-bearing unnatural amino acid, could be displayed in the proper position via co-expression of the DiZPK-specific orthogonal tRNA/aaRS pair. Full-length protrudin protein was detected in all mutants by western blotting with anti-myc monoclonal antibody when 293T cells were cultured in the presence of DiZPK (Supplementary Fig. 2). Depending on the mutated site, expression levels of mutant protrudin corresponded to 10–100% of wild-type protein levels. No protrudin protein was detected if 293T cells were cultured in the absence of DiZPK. Finally, we found that DiZPK failed to be incorporated in seven of the 27 sites, and that the amount of protein synthesized indicated that incorporation efficiency varied from position to position. These results indicate that precise incorporation of DiZPK into protrudin protein is practical and possible by genetic code expansion.

### Detection of native proteins interacting with protrudin

**Figure 2:**
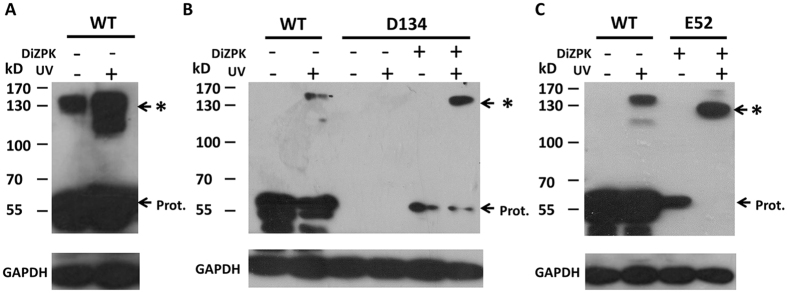
Identification of native proteins that interact with protrudin in HEK293T cell. (**A**) Western blotting analysis of wild type protrudin protein photo-crosslinking. (**B**,**C**) DiZPK-partitioned protrudin photo-crosslinking analysis surveying residues at sites E52 to D134 in protrudin. Asterisks indicate the photo-crosslinked protein complexes.

Following the successful site-specific incorporation of DiZPK into the protrudin protein, we next used the DiZPK probe to capture proteins that interact with protrudin *in vivo*. To that end, HEK293T cells were cultured for 48 h after transfection to express protrudin with DiZPK incorporated at different sites, and photo-crosslinking was triggered by exposing cells to UV light (365 nm) for 10 min. Cells expressing wild-type protrudin (WT-protrudin) subjected to UV light were used as control. The crosslinked proteins of 20 protrudin variants were analyzed by SDS-PAGE and immunoblotting with anti-Myc antibody (Supplementary Fig. 3). Polypeptides denoted by asterisks showed a multiple molecular weight increase corresponding to the formation of SDS-resistant WT-protrudin dimers (Fig. 2A), which is consistent with previous results suggesting that protrudin might perform its function as an oligomer^9^. Moreover, we found that the amount of SDS-resistant WT/protrudin dimer formation is low and difficult to detect with low amounts of sample (Supplementary Fig. 3, Fig. 4A). Furthermore, a protrudin dimer was also obtained after DiZPK-induced photo-crosslinking at site 134 of protrudin, suggesting that residue 134 may be part of the protrudin domain that forms dimers (Fig. 2B). Introduction of DiZPK at position E52 led to a change in molecular weight, suggesting that DiZPK-protrudin captured new interactors (Fig. 2C).

### Identification of protrudin-interacting proteins by co-immunoprecipitation and liquid chromatography-mass spectrometry

**Figure 3:**
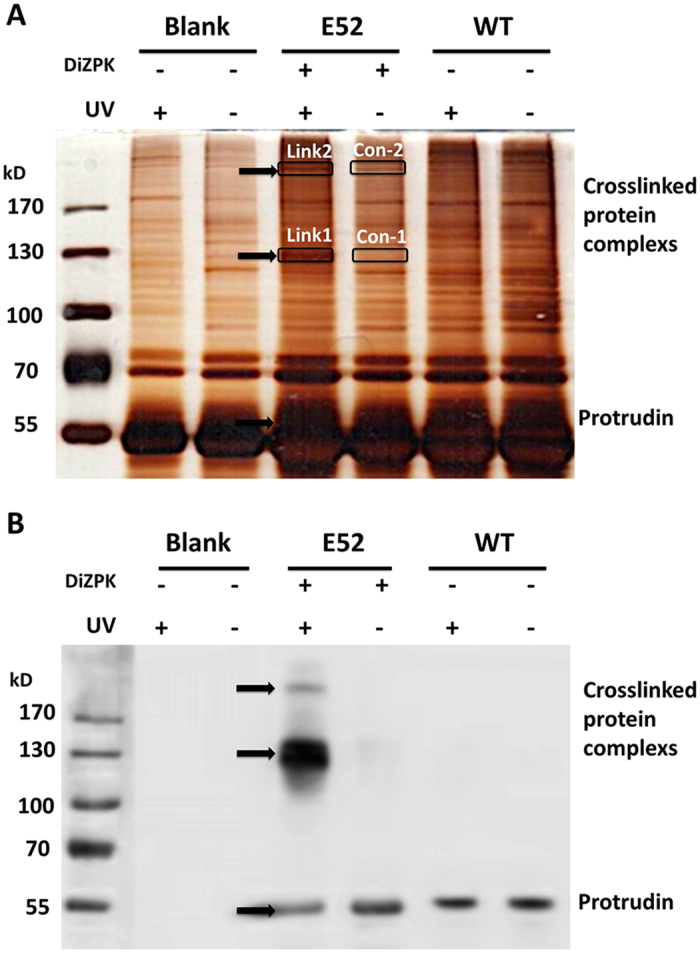
Sliver staining and western blotting analysis of protrudin-DiZPK protein complexes. (**A**) The precipitation products of HEK293T cells were analyzed by SDS-PAGE and by silver staining. (**B**) Analysis of precipitation products by western blotting with anti-myc rabbit polyclonal antibody.

To identify the proteins crosslinked to protrudin at site E52, we expressed protrudin-E52DiZPK in HEK293T cells, induced crosslinking, and enriched protrudin-E52DiZPK protein complexes in cell lysates by co-immunoprecipitation (co-IP) (Fig. 3). The parallel expression of protrudin-E52DiZPK without crosslinking eliminates the possibility to obtain the artificial interactions caused by over-expression of protrudin. Purified proteins were separated by SDS-PAGE and stained with silver (Fig. 3A), and detection of the corresponding proteins (Link1, Link2, indicated by arrows in Fig. 3B) by western blotting confirmed that the purified proteins resulted from the photo-crosslinking reaction between DiZPK-protrudin and its unknown interactors. The stained gels containing Link1, Link2, Con1, and Con2 were then sliced and subjected to trypsin, and the peptides generated were subjected to liquid chromatography (LC) and tandem MS as described in Methods. MS results identified several proteins complexed with protrudin: DNA repair-related proteins, ubiquitin-related proteins, translation-related proteins, and many vesicle-related proteins, none of which has been reported as putative *in vivo* interactors of protrudin. We chose a total of eight proteins (Table S2) that were present in more than one protrudin-E52DiZPK complex but were not recovered from control cells harboring WT-protrudin (Link1-Con1, Link2-Con2). Among the identified proteins, we found, remarkably, extended synaptotagmin-1, ankyrin repeat and FYVE domain-containing protein 1 isoform 1 (ANKFY1), uveal autoantigen with coiled-coil domains and ankyrin repeats isoform 1 (UACA), and FASN. All these potential protrudin client proteins are valuable candidates for further validation. Furthermore, the protein complexes crosslinked to protrudin at site R330, denoted by asterisks in Supplementary Fig. 3E, were also analyzed by LC-MS. KIF5, a known protrudin interactor, was identified, which is consistent with previous results that protrudin perform its interaction with KIF5 through the NH2-terminal portion of the FYVE domain (amino acids 274–361)^25^. It is indicated that we could obtain different interacting proteins by incorporating DiZPK into different sites of protrudin. These results suggest that the method we employed here is practical and reliable.

### Validation of FASN as a novel interactor of protrudin

**Figure 4:**
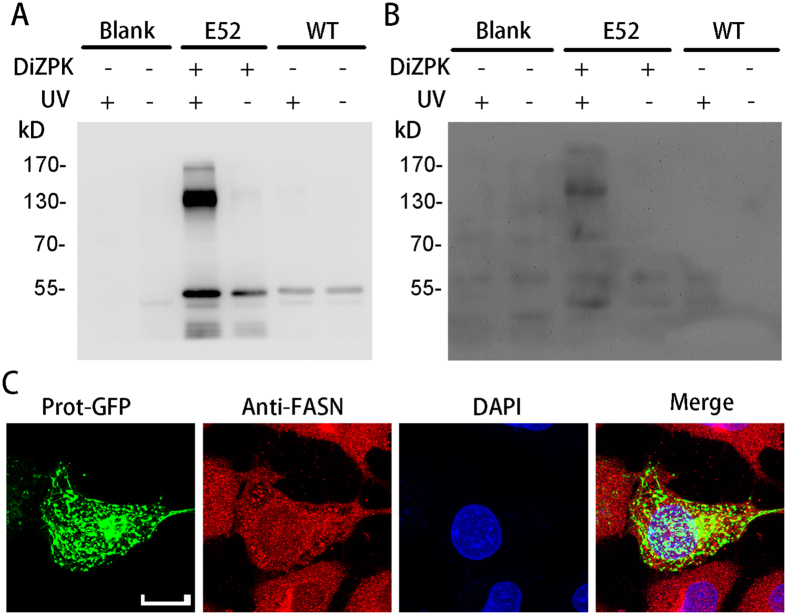
Determination of protein-protein interaction between fatty acid synthase and protrudin. The precipitation products of HEK293T cells were analyzed by SDS-PAGE and by western blotting with anti-myc antibody (**A**) or anti-fatty acid synthase (FASN) antibody (**B**). Co-localization of protrudin and FASN in HeLa cells was analyzed using laser scanning confocal microscope (**C**). Scale bars: 10 μm.

To confirm the interactions between protrudin and its interactors identified by MS, we performed immunoblotting (IB) analysis to confirm the presence of the clients above in the crosslinked complexes. Control 293T cells without any exogenous proteins and 293T cells expressing WT protrudin and the protrudin E52DiZPK mutant were irradiated or not with UV light, and then the cell lysates were enriched and subjected to western blotting with anti-UACA, anti-GSTP, anti-ANKFY1, and anti-FASN antibodies. Of the four candidates, only FASN was detected in the complex, and its apparent molecular weight (Fig. 4B, Supplementary Fig. 4) was similar to that of the complex detected by the anti-c-myc antibody (Fig. 4A). However, no interaction between FASN and protrudin was detected by co-IP without photo-crosslinking. To further confirm the protrudin-FASN interaction, we determined the subcellular localization of FASN and protrudin in HeLa cells, and found that these proteins have a similar (but not the same) distribution throughout the cell body (Fig. 4C, Supplementary Fig. 5). Taken together, these experiments validate FASN as a transient or weak interactor that binds to protrudin in its dynamic transport process.

### Incorporation of DiZPK in protrudin-E52DiZPK does not alter the distribution and function of protrudin

**Figure 5:**
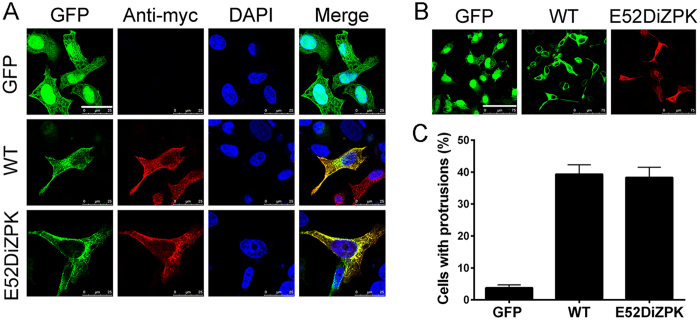
Function and subcellular localization of protrudin-E52DiZPK. (**A**) Co-localization analysis of wild type protrudin and protrudin-E52DiZPK mutant in HeLa cells. Scale bars: 25 μm. (**B**) Qualitative evaluation of protruding-E52DiZPK function in HeLa cells by confocal imaging. (**C**) Quantitative analysis of HeLa cells with protrusions. Data are shown as means ± SD from three independent experiments. **P < 0.01, ***P < 0.001. Scale bars: 75 μm.

To test whether the site-specific incorporation of DiZPK in protrudin-E52DiZPK affects its distribution and function, both aspects of protrudin-E52DiZPK and WT-protrudin were compared. We co-transfected plasmids encoding protrudin-myc, protrudin-E52DiZPK-myc, and protrudin-GFP into HeLa cells. Both wild-type and variant protrudin co-localized with protrudin-GFP (Fig. 5A), indicating that DiZPK incorporation does not alter the distribution of protrudin in cells.

Protrudin overexpressed in HeLa and NIH3T3 cells was distributed both in the endosome and endoplasmic reticulum and, remarkably, induced a directional protrusion extension^1^,^9^. We also observed this phenomenon in 293T cells (Supplementary Fig. 6), indicating that it is a universal phenomenon in non-neuronal cells. We investigated the influence of DiZPK incorporation on the induction of a protrusion extension by protrudin. Comparison of the morphology of HeLa cells harboring plasmids expressing protrudin-GFP or protrudin-E52DiZPK-myc showed no significant difference (Fig. 5B). Moreover, the percentage of cells with protrusions in each group was also similar (Fig. 5C). These results suggest that protrudin-E52DiZPK retained its ability to induce protrusion extensions.

### Fatty acid synthase cooperates with protrudin to regulate membrane outgrowth of cellular protrusion in HeLa cells

**Figure 6:**
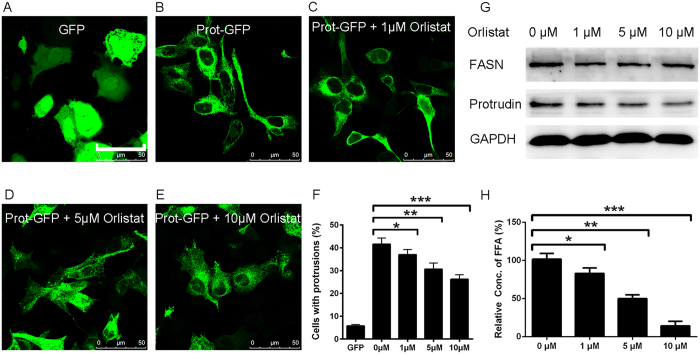
Determination of fatty acid synthesis and protrudin-induced cellular protrusion formation. (**A**–**F**) Cell protrusion formation was quantified in HeLa cells. (**G**) Fatty acid synthase and protrudin protein expression was analyzed by western blotting. (**H**) Free fatty acid concentration was also determined. Quantitation of protrusion formation in HeLa cells treated as in Fig. 5A. Scale bars: 50 μm.

Having identified the protrudin-FASN interaction and the effects of DiZPK on protrudin function, we next investigated whether the protrudin-FASN interaction is required for cellular protrusion extension. We co-transfected plasmids encoding GFP and protrudin-GFP into HeLa cells to induce protrusion formation, and then orlistat, a FASN inhibitor, was added to a final concentration of 1 μM, 5 μM, or 10 μM. After 48 h of treatment, the endogenous protrudin expression levels, the synthesis of free fatty acids (FFAs), and the percentage of HeLa cells with protrusions, all declined in a dose-dependent manner (Fig. 6). These results suggest that the FASN-synthesized FFAs are essential for cellular protrusion outgrowth.

**Figure 7:**
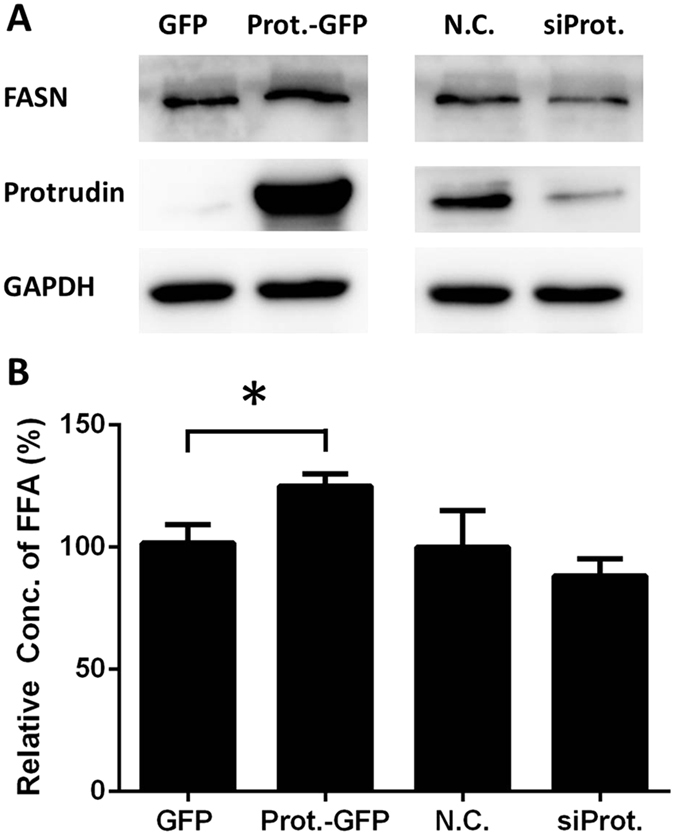
Comparison of protrudin expression and free fatty acid synthesis. (**A**) Immunoblotting analysis of overexpression or repression of protrudin in HeLa cells. (**B**) Free fatty acid concentration of the transfected HeLa cells was determined.

To further study the relation between protrudin and FASN, HeLa cells were transfected with an expression vector containing green fluorescent protein-tagged protrudin (or with the corresponding empty vector) or with the chemically-synthesized siRNA for protrudin (or the negative control siRNA, N.C.) for 48 h, and then both FASN protein expression levels and FFA synthesis were determined (Fig. 7). The results show a positive correlation between protrudin and FASN expression levels in HeLa cells. Moreover, FFA synthesis also followed protrudin levels (Fig. 7B), which indicates that over-expression of protrudin may increase the FFAs synthesis activity of FASN. These results suggest that cellular protrusion extension is highly dependent on the interaction between FASN and protrudin in HeLa cells, and the whole process of protrusion formation is closely related to lipid synthesis.

## Discussion

Biology largely relies on functional interplay of proteins in the crowded and heterogeneous environment inside cells, and functional protein interactions are often weak and transient. Despite their importance, such interactions are exceedingly difficult to identify and characterize in living cells, especially in extreme physiological condition such as low pH. The emerging technique of genetic code expansion, the insertion of a stop codon to encode an amino acid not found among the 20 natural amino acids, exhibits many relevant, promising features^26^. Using a highly efficient photo-crosslinking unnatural amino acid, DiZPK, site-specifically incorporated into a bait protein for capturing unknown PPIs in living cells^22^, we succeeded in identifying the native client proteins of the acidic endosome protein protrudin, which facilitates cellular extension. Further characterization, which focused on FASN, revealed a unique cooperation mechanism between protrudin and FASN in HeLa cells.

FASN is an important enzyme in lipid biosynthesis whose localization may change via interactions with other proteins in the cytosol^27^,^28^. Recently it has been reported that Rab18-mediated membrane trafficking of FASN and NS3 facilitates DENV 33 replication, probably by ensuring a sufficient and coordinated lipid supply for membrane proliferation and arrangement^29^. It has been described that protrudin containing ER-endosome contact sites provides platforms for kinesin-1 loading onto endosomes, and kinesin-1 mediates translocation of endosomes to the cell periphery fuelled by repeated ER contacts, promoting cellular protrusion^30^,^31^. Unlike canonical FYVE family proteins, which bind phosphatidyl-3-phosphate selectively, the protrudin FYVE domain has a binding preference for phosphatidylinositol-3,4-bisphosphate, phosphatidylinositol-4,5-bisphosphate, and phosphatidlyinositol-3,4,5-trisphosphate, which makes protrudin able to bind a wide range of lipid substrates^12^. In our study, inhibition of FFA synthesis suppressed protrudin-induced cellular protrusion extension, and the overexpression of protrudin was also accompanied by increased FFA synthesis. Taking together previous work and our results, we propose that the protrudin-FASN axis may facilitate directional membrane trafficking (Supplementary Fig. 7). Undergoing dynamic transport, protrudin may recruit FASN, which is responsible for the generation of fatty acids. These fatty acids could then be modified to phospholipid and be incorporated into the membrane of the endosome and the endoplasmic reticulum (ER). Protrudin may function together with FASN for lipid synthesis within the tubular ER, which facilitate membrane expansion and increased membrane fluidity conducive to the generation of membrane curvature. With the help of kinesin motors, the membrane-carrying endosomes are transported along microtubules far from the nucleus and into the cell protrusion elongation process. This model is consistent with ER-localized protrudin and location-changing FASN, and requires enzymes from fatty acid metabolic pathways for increased membrane fluidity and remodeling^1^. It is possible that another key protein serves as a bridge to bring protrudin and FASN together and stabilizes the membrane localization of the complex we enriched in HeLa cells. Further work is required to clarify the mechanism of protrudin-related membrane trafficking in cellular protrusion extension.

## Materials and Methods

### Synthesis of the DiZPK photo-crosslinking probe

The photo-crosslinking amino acid DiZPK (Fig. 1A) was synthesized as previously described^22^. Diazirine, the photoactive group of DiZPK, has a superb chemical stability prior to photolysis and photolyzes rapidly when exposed to UV light.

### Construction of expression plasmids

The cDNA encoding human protrudin tagged at its C-terminus with c-Myc was subcloned into pcDNA3.1/Myc-His6 (Invitrogen). The pPylRS/tRNA_CUA_ vector containing the gene encoding the orthogonal amber suppressor aminoacyl-tRNA synthetase/tRNA_CUA_ pairs was supplied by Peng Chen (Chemistry School of Peking University). Site-directed mutagenesis was performed using the Light Quickchange Kit (Stratagene, La Jolla, CA, USA).

### Antibodies and reagents

Anti-Myc (9E10) mouse antibody was obtained from Santa Cruz Biotechnology (Santa Cruz, CA), anti-FASN antibody from abnova (Walnut, CA, USA), anti-protrudin rabbit antibody from Proteintech (Chicago, USA), and orlistat from Sigma-Aldrich (St. Louis, MO, USA).

### Cell culture and transfection

HEK293T cells were cultured under humidified atmosphere at 5% CO_2_ at 37 °C in DMEM (Gibco) supplemented with 10% fetal bovine serum (FBS; Gibco). The cells were transfected using MegaTran1.0 (Origene) and cultured for 48 h after transfection. The photo-crosslinking reaction was triggered by exposing cells expressing protrudin-DiZPK to UV light at 365 nm for 10 min. HeLa cells were cultured under humidified atmosphere of 5% CO_2_ at 37 °C in RPMI 1640 (Gibco) supplemented with 10% fetal bovine serum (FBS; Gibco).

### siRNA transfections

siRNA oligonucleotides were obtained from GenePharma (Shanghai, China). siRNA transfections of HeLa cells were performed using RNAiMax (Invitrogen) according to the manufacturer’s protocol with 20 or 50 nM siRNA oligonucleotide per well. Scrambled RNA was used as negative controls as described previously^32^. The siRNA of protrudin (5′-CAGGUGGCAGAUGCCUUUGUGUU[dT][dT]-3′) has been described previously^30^.

### Preparation of protein, immunoblotting, and immunoprecipitation analyses

Transfected HEK293T cells were lysed by incubation for 15 min at 4 °C with lysis buffer. The lysates were centrifuged at 13,000× *g* for 15 min at 4 °C, and equal amounts of protein from the resulting supernatants were subjected directly to IB or IP. The supernatant was subjected to IP for 2 h at 4 °C with anti-c-Myc antibody and protein A/G-Sepharose (Santa Cruz, CA). The immunoprecipitate was washed five times with lysis buffer and then subjected to silver staining and IB. The images were scanned with UVP imager (UVP, LLC, CA, USA).

### Protein identification by liquid chromatography-mass spectrometry analysis

The affinity-purified protein complexes were concentrated by precipitation with chloroform and methanol, fractionated by SDS-PAGE, and stained with silver. The stained gel was sliced into 10 equal pieces per lane, and the proteins therein were subjected to in-gel digestion with trypsin. The resulting peptides were dried, dissolved in a mixture of 0.1% trifluoroacetic acid and 2% acetonitrile, and then applied to a nanoflow LC system (Paradigm MS4; Michrom BioResources, Auburn, CA) equipped with an L-column (C18, 0.15 × 50 mm, particle size of 3 μm; CERI, Tokyo, Japan). The peptides were fractionated with a linear gradient of solvent A (2% acetonitrile and 0.1% formic acid in water) and solvent B (90% acetonitrile and 0.1% formic acid in water), with 0–45% solvent B for 20 min, 45–95% for 5 min, and 95–5% for 1 min at a flow rate of 1 μL/min. Eluted peptides were sprayed directly into a Finnigan LTQ mass spectrometer (Thermo Fisher Scientific, San Jose, CA). MS and MS/MS spectra were obtained automatically in a data-dependent scan mode with a dynamic exclusion option. All MS/MS spectra were compared with human protein sequences in the International Protein Index (IPI; European Bioinformatics Institute, Hinxton, United Kingdom) version 3.44 with the use of the MASCOT algorithm. Trypsin was selected as the hydrolytic enzyme, the allowed number of missed cleavages was set to one, and carbamidomethylation of cysteine was selected as a fixed modification. Oxidized methionine and N-terminal pyroglutaminate were searched as variable modifications. Tolerance of MS/MS ions was 0.8 Da. High-scoring peptide sequences (MASCOT score ≥ 20) were considered for correct identification. Identified peptides from independent experiments were integrated and regrouped by IPI accession number.

### Immunofluorescence

Cells were grown on glass coverslips, fixed with 4% paraformaldehyde in phosphate-buffered saline (pH 7.4) (PBS) for 15 min, permeabilized in PBS with 0.5% Triton X-100 for 10 min, and blocked with 3% bovine serum albumin in PBS for 60 min. The cells were then incubated with primary antibodies for 3 h, followed by incubation with FITC or rhodamine-conjugated secondary antibodies (Zhongshan Biotech, China) for an additional 40 min. The nuclei were counterstained with 4, 6-diamidino-2-phenyl-indole (DAPI, Sigma). Images were obtained with a Confocal Laser-Scanning Microscope (Leica TCS SP8, Leica Microsystems, Mannheim, Germany).

### Cell protrusion analysis

Cells with processes with length greater than the longest diameter of the nucleus were counted, and the ratio of the number of these cells to the total number of cells overexpressing protrudin was determined. At least 450 cells were counted per experiment.

### Determination of cellular free fatty acid concentration

Approximately 5 × 10^6^ HeLa cells transfected with protrudin-GFP or siRNA of protrudin and incubated with different concentrations of orlistat were harvested at 48 h after treatment. FFA concentration was determined using the human free fatty acid test kit (Jiancheng Biotech, Nanjing, China).

### Statistical analysis

All experiments were repeated at least three times. Data are shown as means ± standard deviations (SDs). The results were analyzed by one-way ANOVA or Student’s t-tests. Differences with *P* values of less than 0.05 were considered statistically significant.

## Additional Information

**How to cite this article**: Zhang, C. *et al*. Fatty acid synthase cooperates with protrudin to facilitate membrane outgrowth of cellular protrusions. *Sci. Rep.* **7**, 46569; doi: 10.1038/srep46569 (2017).

**Publisher's note:** Springer Nature remains neutral with regard to jurisdictional claims in published maps and institutional affiliations.

## Supplementary Material

Supplementary Information
