# Supplementary material for: Fatty acid synthase cooperates with protrudin to facilitate membrane outgrowth of cellular protrusions
Source: Sci Rep. 2017 Apr 21;7:46569. doi: 10.1038/srep46569 (PMC5399442; doi:10.1038/srep46569)
Supplement: Supplementary Information [file srep46569-s1.doc]

**SUPPLEMENTARY INFORMATION**

**Fatty acid synthase cooperates with protrudin to facilitate membrane outgrowth of cellular protrusions**

Chuanling Zhang^†§*^, Jiaqi Lu^†§^, Huizhong Su^†^, Jing Yang^†^, and Demin Zhou ^†,*^

^†^State Key Laboratory of Natural and Biomimetic Drugs, School of Pharmaceutical Sciences, Peking University, Beijing 100191, China.

^*^Address correspondence to:

Dr. Chuanling Zhang and Prof. Demin Zhou

School of Pharmaceutical Sciences

Peking University

No.38, Xueyuan Road, Beijing 100191, China

Tel: 86-10-8280-5857

Fax: 86-10-8280-5519

Email: zhangchuanling@bjmu.edu.cn, deminzhou@bjmu.edu.cn

^§^ These authors contributed equally to this paper.

**Materials and methods**

**Antibodies:**

Anti-UACA rabbit polyclonal antibody (ab84478) was obtained from abcam (Cambridge, UK), anti-ANKFY1 goat polyclonal antibody (sc-160136) from Santa Cruz Biotechnology (Santa Cruz, CA), and anti-GSTP antibody (ab106268) from abcam (Cambridge, UK). Plasmids pCMV-UACA-GFP and pCMV-ANKFY1-GFP were constructed in our lab.

Table S1. Primer sequences used for mutagenesis studies.

| **Mutation**  **Site** | **Primer** | **Sequence** |
| --- | --- | --- |
| L13 | For | GGGAGTGGGCCGGAGTAGAGCCCCAGCGTGATGCCC |
| L13 | Rev | GGGCATCACGCTGGGGCTCTACTCCGGCCCACTCCC |
| I49 | For | GGTTCTCTCCTACAAGAGGCTGGAGTAGTACCTGGAACCC |
| I49 | Rev | GGGTTCCAGGTACTACTCCAGCCTCTTGTAGGAGAGAACC |
| E52 | For | GGCTGGAGATCTACCTGTAGCCCTTGAAGGATGCAGG |
| E52 | Rev | CCTGCATCCTTCAAGGGCTACAGGTAGATCTCCAGCC |
| D56 | For | CCTGGAACCCTTGAAGTAGGCAGGTGATGGTGTTCG |
| D56 | Rev | CGAACACCATCACCTGCCTACTTCAAGGGTTCCAGG |
| V61 | For | GGATGCAGGTGATGGTTAGCGATACTTGCTCAGGTGGC |
| V61 | Rev | GCCACCTGAGCAAGTATCGCTAACCATCACCTGCATCC |
| D134 | For | GCGTGAGGCAGGAGTAGCTGCAGAGAGTTCGCC |
| D134 | Rev | GGCGAACTCTCTGCAGCTACTCCTGCCTCACGC |
| G191 | For | CTATGGGGCTCTTCTGTAGACAGTCTGCATGCTGTATTTGCTGC |
| G191 | Rev | GCAGCAAATACAGCATGCAGACTGTCTACAGAAGAGCCCCATAG |
| P200 | For | GTCTGCATGCTGTATTTGCTGTAGCTCTGCTGGGTTCTCACCC |
| P200 | Rev | GGGTGAGAACCCAGCAGAGCTACAGCAAATACAGCATGCAGAC |
| N209 | For | GGGTTCTCACCCTTTTATAGAGCACGCTCTTTCTGGGG |
| N209 | Rev | CCCCAGAAAGAGCGTGCTCTATAAAAGGGTGAGAACCC |
| Y226 | For | CCGAGTTGTGTCTGAGTAGAGGGCATCTCTGCAGC |
| Y226 | Rev | GCTGCAGAGATGCCCTCTACTCAGACACAACTCGG |
| T265 | For | GCACGCCTGCCCTCTAGCCCACGGAGGACC |
| T265 | Rev | GGTCCTCCGTGGGCTAGAGGGCAGGCGTGC |
| T271 | For | CCCACGGAGGACCTCTAGCCGGGCAGCGTGGAGG |
| T271 | Rev | CCTCCACGCTGCCCGGCTAGAGGTCCTCCGTGGG |
| S274 | For | CCTCACACCGGGCTAGGTGGAGGAGGCTGAGG |
| S274 | Rev | CCTCAGCCTCCTCCACCTAGCCCGGTGTGAGG |
| E286 | For | GGCTGAGCCAGATGAATAGTTTAAAGATGCGATTGAGGAGG |
| E286 | Rev | CCTCCTCAATCGCATCTTTAAACTATTCATCTGGCTCAGCC |
| F287 | For | GCTGAGCCAGATGAAGAGTAGAAAGATGCGATTGAGGAGG |
| F287 | Rev | CCTCCTCAATCGCATCTTTCTACTCTTCATCTGGCTCAGC |
| D289 | For | GCCAGATGAAGAGTTTAAATAGGCGATTGAGGAGGATGATGAGG |
| D289 | Rev | CCTCATCATCCTCCTCAATCGCCTATTTAAACTCTTCATCTGGC |
| E292 | For | GAGTTTAAAGATGCGATTTAGGAGGATGATGAGGGCGC |
| E292 | Rev | GCGCCCTCATCATCCTCCTAAATCGCATCTTTAAACTC |
| D294 | For | GCGATTGAGGAGTAGGATGAGGGCGCCCC |
| D294 | Rev | GGGGCGCCCTCATCCTACTCCTCAATCGC |
| R330 | For | CGGCTCACGGAGTAGCTCCGCAAGCGC |
| R330 | Rev | GCGCTTGCGGAGCTACTCCGTGAGCCG |
| T349 | For | GCACGGGCTGCTCGGCCTAGTTCTCAGTGCTGAAG |
| T349 | Rev | CTTCAGCACTGAGAACTAGGCCGAGCAGCCCGTGC |
| V352 | For | GCTCGGCCACCTTCTCATAGCTGAAGAAGAGGC |
| V352 | Rev | GCCTCTTCTTCAGCTATGAGAAGGTGGCCGAGC |
| S358 | For | CAGTGCTGAAGAAGAGGCGGTAGTGCAGTAATTGTGGAAACAGC |
| S358 | Rev | GCTGTTTCCACAATTACTGCACTACCGCCTCTTCTTCAGCACTG |
| R369 | For | GGAAACAGCTTCTGCTCTTAGTGCTGCTCCTTCAAGGTGCCC |
| R369 | Rev | GGGCACCTTGAAGGAGCAGCACTAAGAGCAGAAGCTGTTTCC |
| G381 | For | CCCAAGTCCTCCATGTAGGCCACAGCCCC |
| G381 | Rev | GGGGCTGTGGCCTACATGGAGGACTTGGG |
| T383 | For | CCTCCATGGGGGCCTAGGCCCCTGAAGCCC |
| T383 | Rev | GGGCTTCAGGGGCCTAGGCCCCCATGGAGG |
| E386 | For | GGCCACAGCCCCTTAGGCCCAGAGGGAGACTG |
| E386 | Rev | CAGTCTCCCTCTGGGCCTAAGGGGCTGTGGCC |
| F393 | For | CCAGAGGGAGACTGTGTAGGTGTGTGCCTCG |
| F393 | Rev | CGAGGCACACACCTACACAGTCTCCCTCTGG |

**Table S2. Summary of identified protrudin clients.**


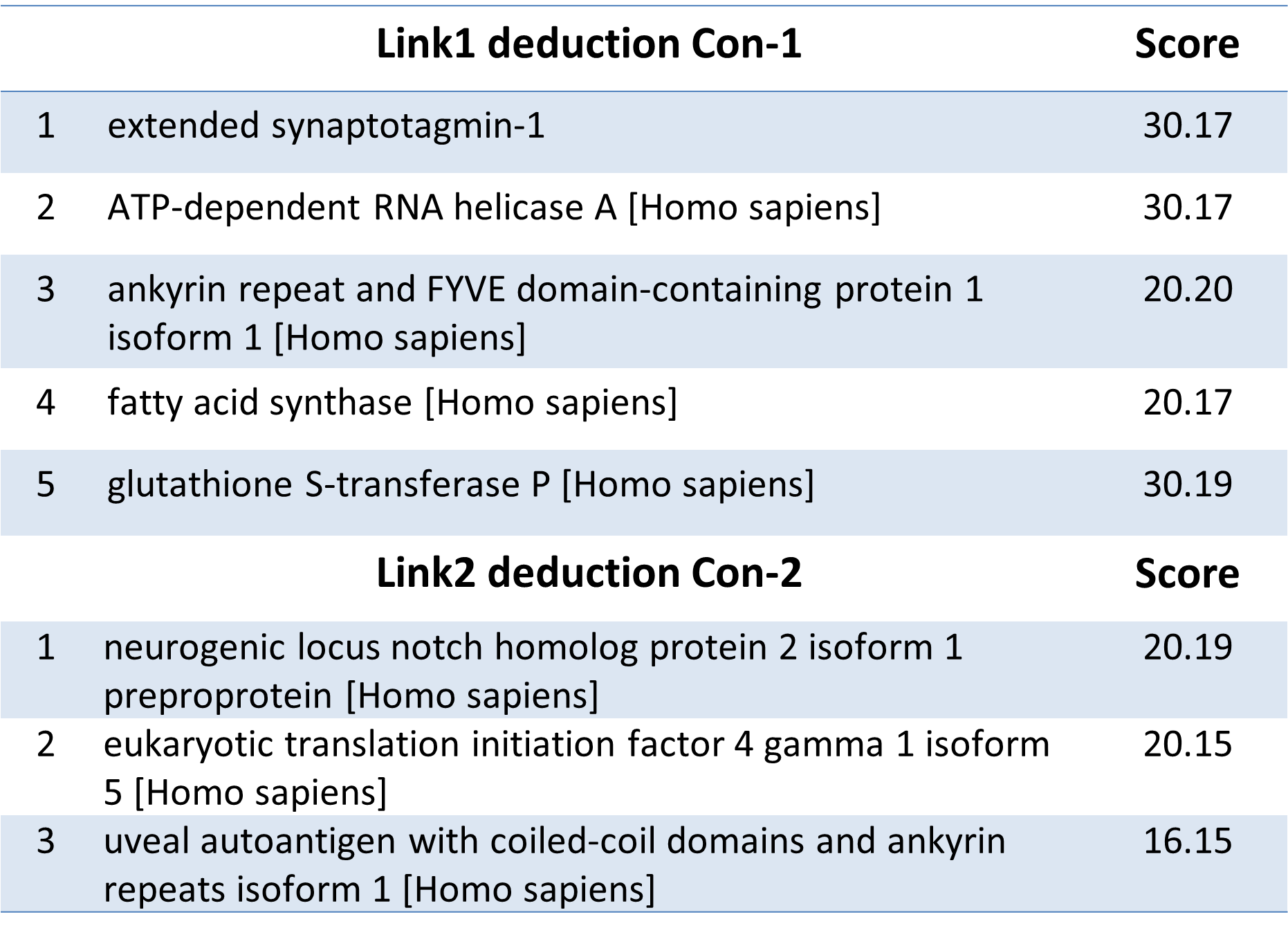


The protein scores from one of two independent experiments are shown, and all scores have a P-value <0.05.


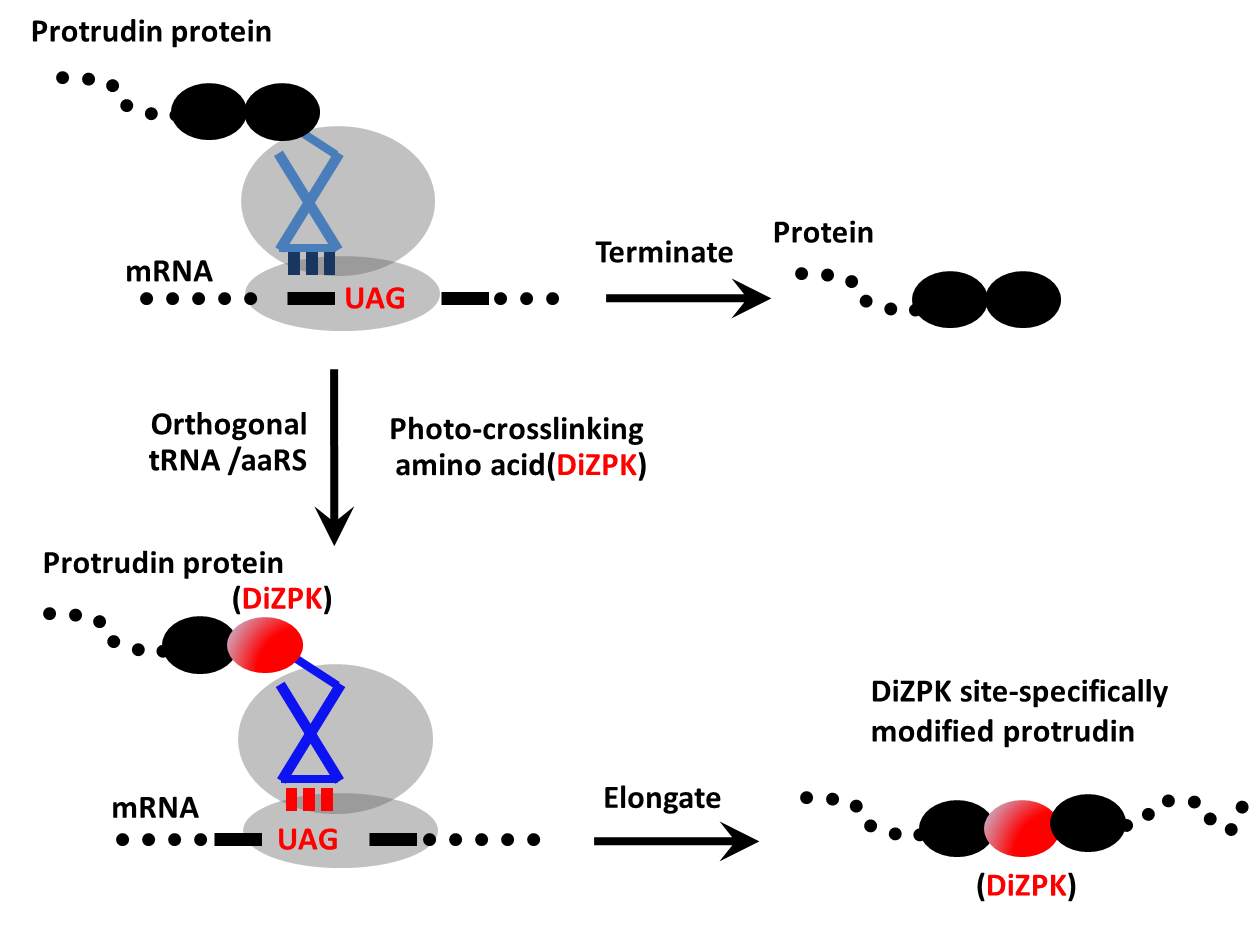


**Supplementary Fig. 1.** Schematic representation of genetic code expansion for site-specific incorporation of DiZPKs. The DiZPKs were incorporated into protrudin protein via UAG-code and an orthogonal aaRS/tRNA.


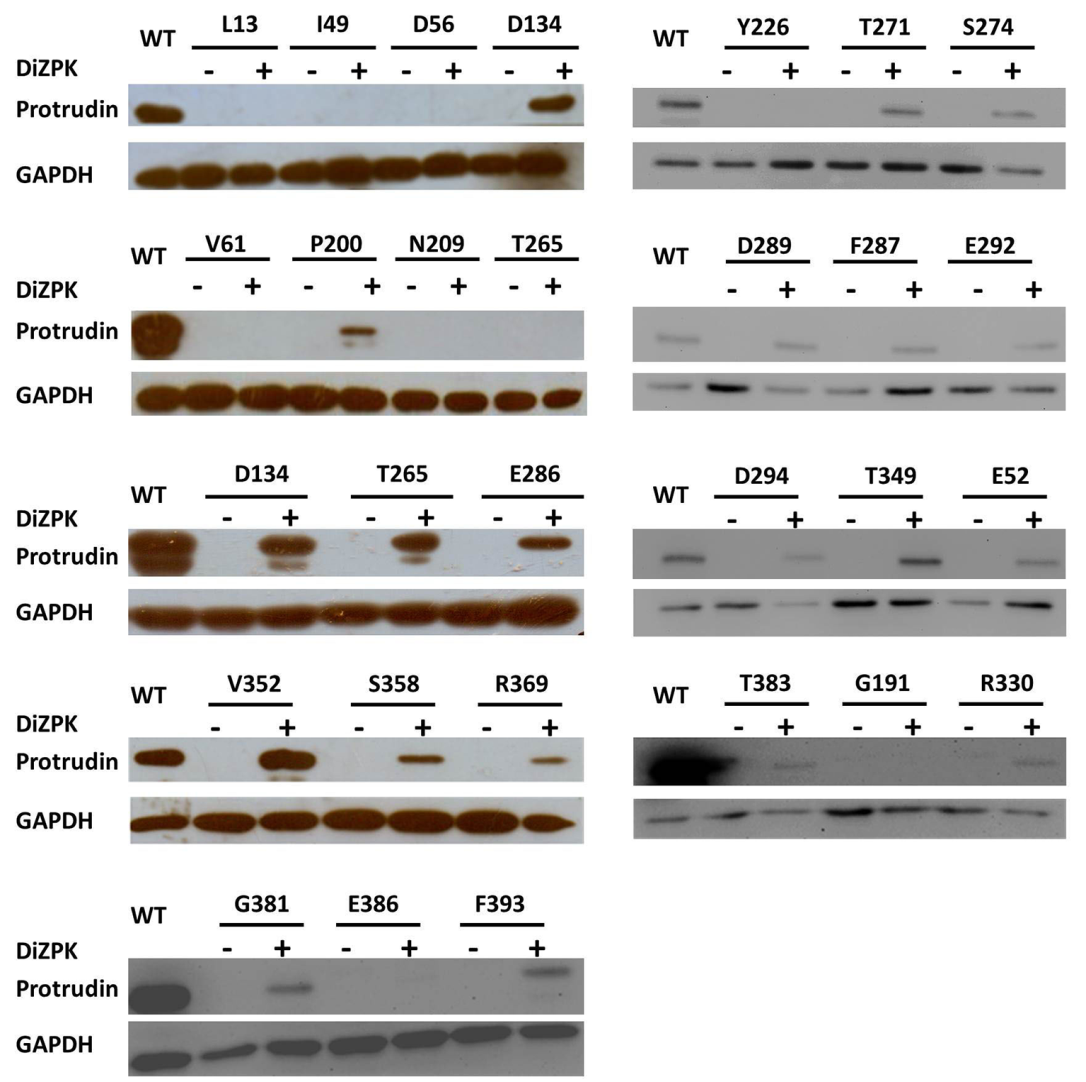


**Supplementary Fig. 2.** Immunoblotting analysis with anti-Myc antibody showing the incorporation of DiZPK into protrudin-Myc variants bearing a single amber mutation at each indicated site with (+) and without (-) 1 mM DiZPK.


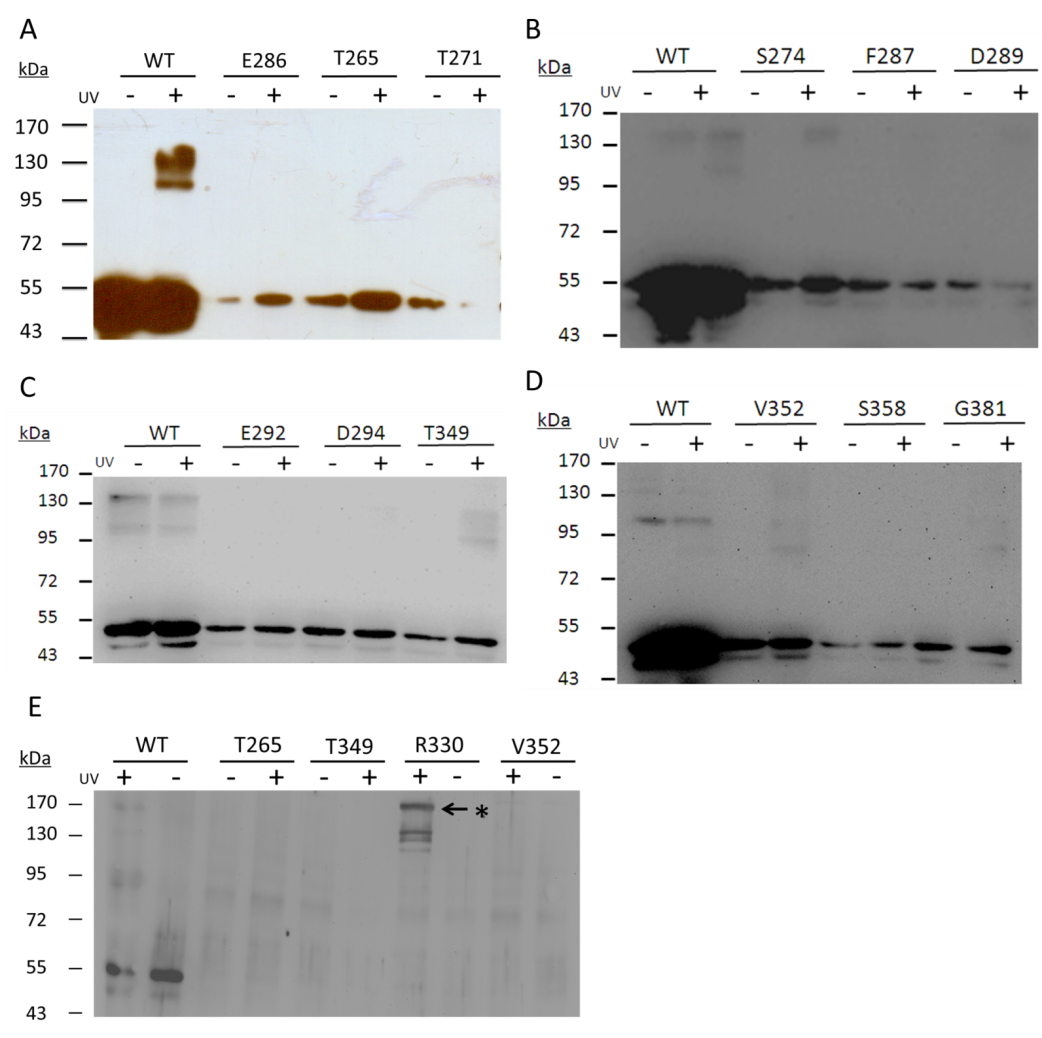


**Supplementary Fig. 3. Identification of native protein interactions with protrudin. a**-**e**, The 293T cells expressing protrudin-Myc with DiZPK incorporated at different sites were irradiated with UV light (365 nm) for 10 min, and then the protein lysates were obtained and subjected to immunoblotting with anti-Myc antibody.


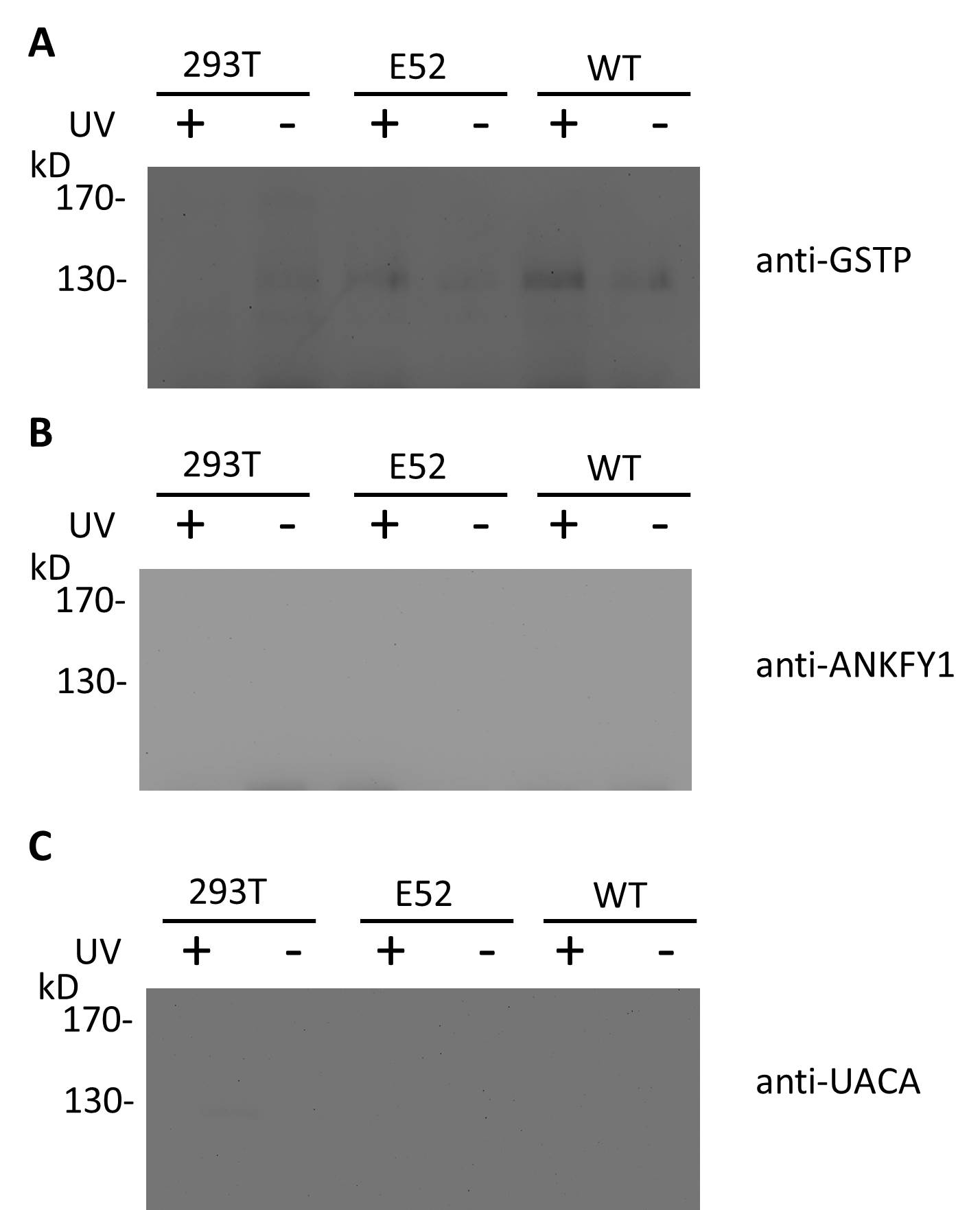


**Supplementary Fig. 4 Western blotting analysis of protrudin-DiZPK protein complexes.** HEK293T cells expressing protrudin-E52DiZPK-myc or WT-protrudin-myc with or without DiZPK or UV irradiation were harvested and co-immunoprecipitated using anti-myc mouse monoclonal antibody. The precipitation products were analyzed by western blotting using anti-GSTP antibody (A), anti-ANKFY1 goat polyclonal antibody (B), and anti-UACA rabbit polyclonal antibody.


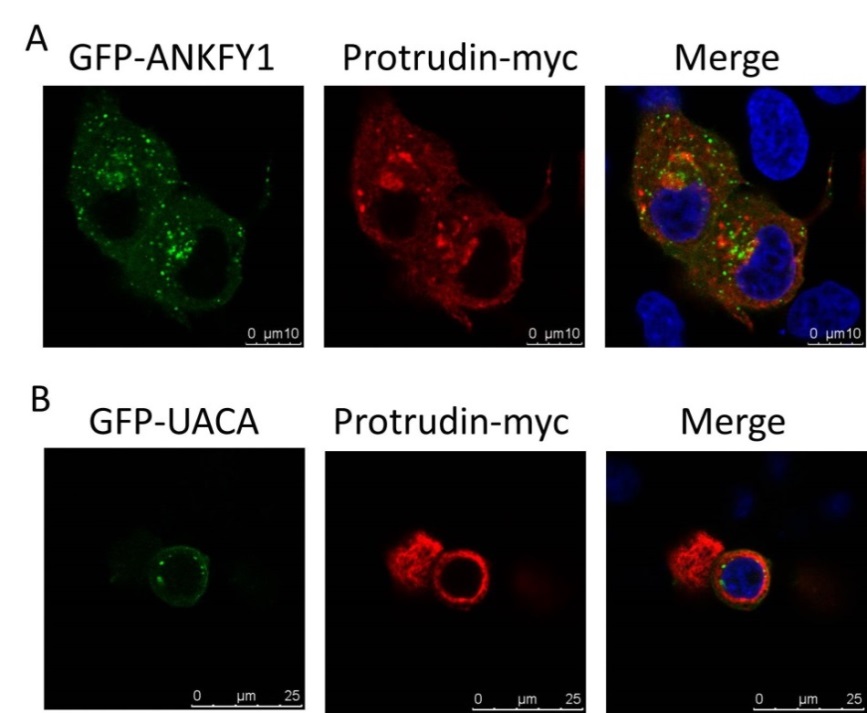


**Supplementary Fig. 5. Co-localization analysis of protrudin, ANKFY1, and UACA.** Protrudin-myc-containing plasmid was co-transfected with GFP-ANKYF1- or GFP-UACA-containing plasmids into HeLa cells, and after 30 h the cells were fixed and immunolabeled using anti-fatty acid synthase primary antibody and Alexa555-labeled secondary antibody. The co-localization of protrudin with ANKFY1 (A) and UACA (B) was analyzed using laser scanning confocal microscopy. Scale bars are 10 μm in A and 25 μm in B.


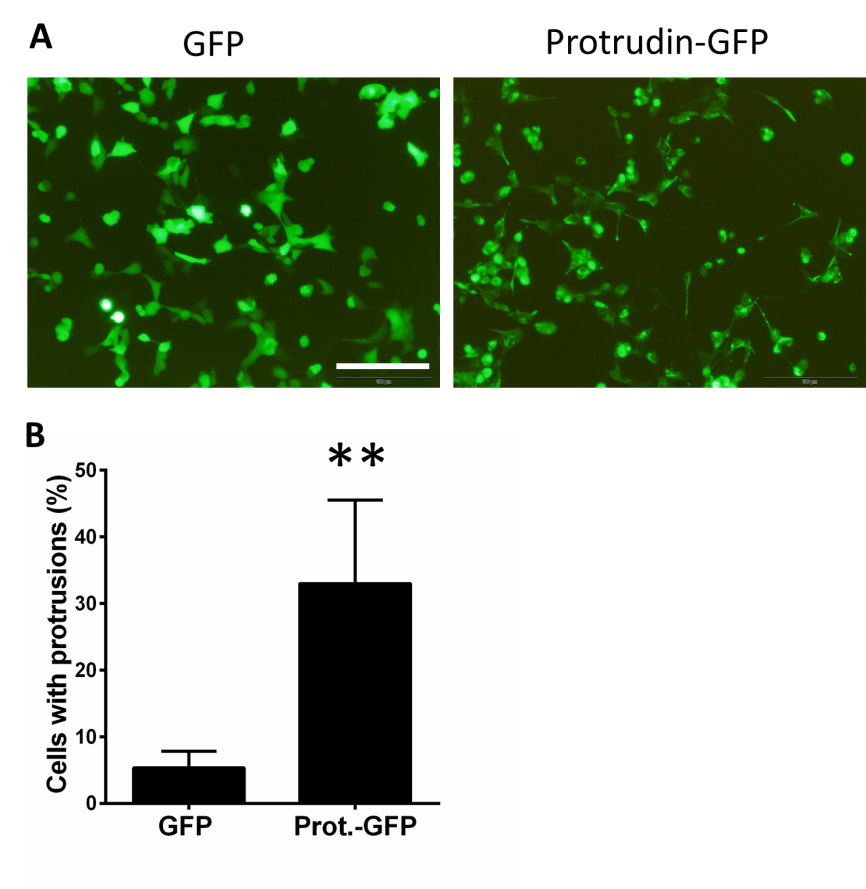


**Supplementary Fig. 6. Protrudin induces outgrowth of cellular protrusion.**

A, HEK293T cells were transfected with protrudin-GFP, and the fluorescence was monitored by fluorescence microscope. Scale bars are 100 μm.

B, Cells with processes with length greater than the longest diameter of the nucleus were counted, and the ratio of the number of these cells to the total number of cells overexpressing protrudin was determined. At least 400 cells were counted per experiment. Data are shown means ± SD from three independent experiments. ***P* < 0.01.


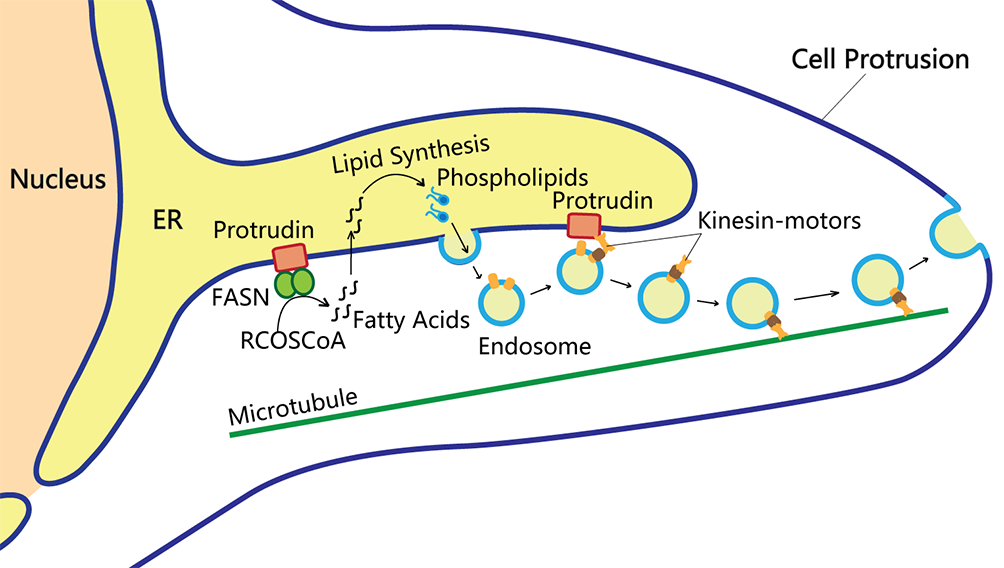


**Supplementary Fig. 7. A fatty acid synthase-protrudin cooperation model for protrudin-promoted cellular protrusion.** Protrudin interacts with FASN during cellular protrusion, which requires large amounts of lipids for membrane extension. FASN catalyzes the synthesis of long-chain fatty acids from RCOSCoA. Locally synthesized fatty acids are delivered to the ER and combine with glycerol-3-phosphate to form phospholipids. New phospholipids are added to the ER membrane, and then travel from the ER to endosome vesicles. Protrudin may drive the kinesin-dependent transport of endosome vesicles along microtubules far from the nucleus to the cell protrusion elongation process. FASN, fatty acid synthase; ER, endoplasmic reticulum; RCOSCoA, malonyl-CoA and acetyl-CoA.
